# Role of the Non-Canonical RNAi Pathway in the Antifungal Resistance and Virulence of Mucorales

**DOI:** 10.3390/genes12040586

**Published:** 2021-04-17

**Authors:** José Tomás Cánovas-Márquez, María Isabel Navarro-Mendoza, Carlos Pérez-Arques, Carlos Lax, Ghizlane Tahiri, José Antonio Pérez-Ruiz, Damaris Lorenzo-Gutiérrez, Silvia Calo, Sergio López-García, Eusebio Navarro, Francisco Esteban Nicolás, Victoriano Garre, Laura Murcia

**Affiliations:** 1 Departamento de Genética y Microbiología, Facultad de Biología, Universidad de Murcia, 30100 Murcia, Spain; josetomas.canovas@um.es (J.T.C.-M.); carlos.lax@um.es (C.L.); ghizlane.tahiri@um.es (G.T.); joseantonio.perez6@um.es (J.A.P.-R.); damaris.lorenzo@um.es (D.L.-G.); slg5@um.es (S.L.-G.); sebi@um.es (E.N.); fnicolas@um.es (F.E.N.); vgarre@um.es (V.G.); 2 Department of Molecular Genetics and Microbiology, Duke University Medical Center, Durham, NC 27710, USA; mariaisabel.navarro3@um.es (M.I.N.-M.); carlos.perez6@um.es (C.P.-A.); 3 School of Natural and Exact Sciences, Pontificia Universidad Católica Madre y Maestra, Santiago de los Caballeros 51033, Dominican Republic; s.calo@ce.pucmm.edu.do

**Keywords:** virulence, antifungal resistance, non-canonical RNAi, epimutant, R3B2, RdRP, transposon, genome stability, Mucorales, mucormycosis

## Abstract

Mucorales are the causal agents for the lethal disease known as mucormycosis. Mortality rates of mucormycosis can reach up to 90%, due to the mucoralean antifungal drug resistance and the lack of effective therapies. A concerning urgency among the medical and scientific community claims to find targets for the development of new treatments. Here, we reviewed different studies describing the role and machinery of a novel non-canonical RNAi pathway (NCRIP) only conserved in Mucorales. Its non-canonical features are the independence of Dicer and Argonaute proteins. Conversely, NCRIP relies on RNA-dependent RNA Polymerases (RdRP) and an atypical ribonuclease III (RNase III). NCRIP regulates the expression of mRNAs by degrading them in a specific manner. Its mechanism binds dsRNA but only cuts ssRNA. NCRIP exhibits a diversity of functional roles. It represses the epimutational pathway and the lack of NCRIP increases the generation of drug resistant strains. NCRIP also regulates the control of retrotransposons expression, playing an essential role in genome stability. Finally, NCRIP regulates the response during phagocytosis, affecting the multifactorial process of virulence. These critical NCRIP roles in virulence and antifungal drug resistance, along with its exclusive presence in Mucorales, mark this pathway as a promising target to fight against mucormycosis.

## 1. Introduction

The study of the processes regulating antifungal drug resistance and virulence of Mucorales is important because these fungi are the causal agents for the lethal infectious disease known as mucormycosis. Patients with mucormycosis have a poor prognosis as their mortality rates can reach 90% when the infection disseminates through the bloodstream [1,2]. The main reason explaining this high mortality rate is the lack of effective antifungal treatments. Mucorales show an innate high resistance to most of the antifungal compounds currently used in clinical settings [3,4,5]. Besides the availability of potent and efficient new antifungal compounds, most of the current treatments for mucormycosis infections are based on lipidic formulations of amphotericin B. This compound shows antifungal activity against Mucorales, but numerous secondary effects prevent its prolonged use in many patients [6,7,8]. The antifungal drug resistance of Mucorales is the main problem to treat this infection, which is encouraging researchers to focus their investigation on the discovery of new genes, pathways, methodologies, and virulence factors that can be useful to design effective antifungal compounds [9,10,11,12,13,14,15,16,17,18].

**Figure 1:**
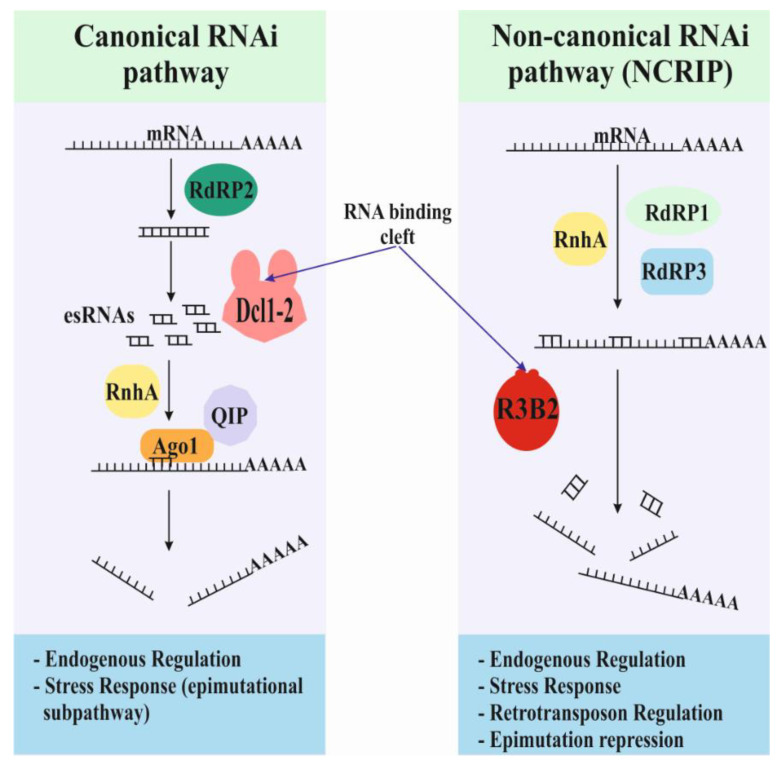
Models and functions for the two endogenous RNAi-based pathways. There are two endogenous RNAi-based pathways in Mucorales, the canonical and non-canonical pathways. The canonical pathway uses the conserved core machinery based on the activities of Dicer and Argonaute proteins. The non-canonical pathway relies on a new RNase type III: the R3B2 protein. R3B2 binds double-strand RNA (dsRNA) but only cuts single-strand RNA (ssRNA).

The innate antifungal drug resistance and virulence observed in Mucorales must be based on specific mechanisms not found in other fungi [4,19,20]. Among these mechanisms, the most studied and related to virulence and antifungal drug resistance is the RNAi mechanism. The first RNAi-based mechanism discovered in Mucorales was the exogenously induced RNAi pathway, a defense system against transgenes and plasmids. Later, two endogenous RNAi pathways were discovered: the canonical and the non-canonical pathways (Figure 1) [15,21,22,23,24,25]. A subtype of the canonical pathway, the epimutational pathway, is directly involved in the generation of transient antifungal drug resistances. This pathway is capable of silencing the gene that encodes the target proteins inhibited by the antifungal compound [19]. In the absence of the target protein, the antifungal compound is unable of inhibiting the regular growth of the fungus. The non-canonical RNA interference pathway (NCRIP) was initially described as a non-specific RNA degradation mechanism, although further studies demonstrated its role in regulating several processes [23,26,27].

In this review, we will focus on the discovery of NCRIP, its components and machinery mechanism, and its role in regulating virulence in Mucorales. Moreover, we will examine the interaction between NCRIP and the epimutational pathway, as several studies demonstrated the negative regulating function of the first over the second, establishing the role of NCRIP in the regulation of the antifungal drug resistance mechanism [19,26].

## 2. Discovery of the Non-Canonical RNA Interference Pathway (NCRIP)

Since the discovery of RNAi in iconic models such as *Petunia hybrida*, *Neurospora crassa*, and *Caenorhabditis elegans* and the discovery of its role in the genome defense against viruses and transposons, the RNAi world has grown with numerous branches showing different pathways involved in the endogenous regulation [24,28,29,30]. A wide variety of endogenous small RNAs (esRNAs) has been described across the Eukarya domain, showing distinct functional roles in the regulation of gene expression [24,31]. There is a canonical common machinery core in the biogenesis of most of these regulatory esRNAs. This is a conserved machinery consisting of two fundamental enzymatic activities. The first one is a Dicer protein (an RNase type III) responsible for the processing of double-stranded RNA (dsRNA) that generates the effectors of the pathway: the small RNAs (sRNAs). The second one is the Argonaute protein that harbors the main functional activities of the RNA-induced silencing complex (RISC) [32]. The Argonaute role in the RISC is the interaction with the guide strands of the sRNAs to use them to localize the target transcripts and repress their expression [33,34,35]. In a group of eukaryotic organisms, including plants, fungi and nematodes, there is another essential component, the RdRPswas, which is responsible for generating the dsRNA and the amplification of the signal by generating a feedback loop [36,37].

Aside from the canonical RNAi pathways, several other alternative and non-canonical pathways have been discovered, in which the central role of Dicer and Argonaute has been substituted by other exonucleases and Argonaute-like proteins. These non-canonical pathways produce the Piwi-interacting RNAs (piRNAs), some unique miRNAs, and miRNA-like (milRNA) [38,39,40,41]. In these cases, the catalytic activity of Argonaute family proteins and the trimming activity of specific exonucleases are required to produce mature esRNAs. Most non-canonical esRNAs are poorly conserved, and their functional role is widely unknown.

Among Mucorales, the fungus *Mucor lusitanicus* has served as a unique model to identify both canonical and non-canonical esRNAs and study their different functional roles (previously known as *Mucor circinelloides* f. *lusitanicus,* [42]) [21,23,43]. An initial thorough study identified several classes of canonical esRNAs (specifically called ex-siRNAs because of their exon targeting activity) [22]. Further studies found a diverse variety of functional roles associated with these ex-siRNAs [43]. The same initial study described the first evidence of a non-canonical RNAi pathway in this fungus, although this study mainly focused on the canonical esRNAs [22]. Later, a second study in *M. lusitanicus* deepened in the dissection of this new non-canonical RNAi pathway [23]. This second study specifically analyzed the *rdrp*-dependent and *dicer*-independent esRNAs found in the previous study. Massive sequencing of the small RNome in the mutants *dicer-*, *rdrp-1-*, and *rdrp-2-* discovered new loci producing sRNAs in a *dicer*-independent but *rdrp-1-* and *rdrp-2*-dependent manner. The newly identified sRNAs displayed three main differences compared to the canonical esRNAs. Thus, two of these differences were that they mostly had the same strand sense that mRNAs and did not show only one size as the canonical esRNAs, but rather a range of different sizes. These results suggested that this newly discovered *rdrp-*dependent and *dicer-*independent pathway was likely involved in a new RNA degradation pathway that was partially based on the RNAi machinery. Finally, the third specific feature of these non-canonical sRNA was a strong bias for uracil in the penultimate position of the 3′ end, and another strong bias for uracil two nucleotides upstream of the 5′ end. In contrast, canonical esRNAs show a specific preference for uracil at the 5′ end [22]. This particular structure of the non-canonical sRNAs indicated a non-random degradation of the mRNA, suggesting the existence of a specific RNase activity in the biogenesis pathway.

Another result of the initial study of NCRIP showed that this pathway could regulate the gene expression of target mRNAs [23]. The specific structure of the non-canonical sRNAs and the fact that these sRNAs are lost in the *rdrp* mutants suggested the regulatory role of NCRIP. The accumulation of target mRNAs was analyzed in *rdrp* mutants compared to the wild type and dicer mutants. These analyses showed that the lack of NCRIP activity resulted in a considerable increase of its target gene expression. These results and the specific structure of the non-canonical sRNAs indicated that NCRIP was not a common RNA degradation pathway but rather a new RNA-expression regulatory mechanism [23].

Once the non-canonical pathway was identified and characterized, the main question to identify the unknown enzyme in charge of cutting the target mRNAs and substituting the activity of the canonical Dicer proteins. Thus, to find the new RNase, an in silico analysis of *M. lusitanicus* genome resulted in twenty-four proteins with putative endoribonuclease activity. Among them, four candidates were selected for the generation of knockout mutants. This selection accounted for undescribed candidates not previously involved in the RNAi or other well-known RNA degradation pathways. The genetic analysis of these four mutants concluded that only one of them resulted in the lack of NCRIP activity [23]. This mutant was a knockout in a gene coding for a protein containing an RNaseIII domain (r3) and two dsRNA binding domains (b2), which determined the name of the corresponding gene as *r3b2*. Mutants in this gene also showed an increase in the expression of the NCRIP target mRNAs. Moreover, a new deep sequencing of the sRNome in these mutants showed the corresponding lack of non-canonical sRNAs. Identifying the R3B2 protein and the characterization of its role in the biogenesis of the non-canonical sRNAs definitively indicated that this new RNase was part of the NCRIP mechanism [23].

## 3. Structural and Functional Analysis of R3B2

The characterization of NCRIP and its degradation products in the Dicer null mutants led to the discovery of the atypical RNase R3B2 in *M. lusitanicus* [23]. This RNase shows an RNase III-like domain (RIIID) together with two double-strand RNA binding domains (dsRBD), which is an unusual feature for a protein with a hypothesized activity against single-stranded RNA (ssRNA). In fact, both analyses in vivo by the yeast two-hybrid interaction assay and in vitro by size exclusion chromatography and static light scattering indicate that R3B2 dimerizes through the RIIID [44]. Likewise, bacterial RNase III and the two RIIIDs of eukaryotic Dicer and Drosha type RNases III dimerizes to cut each strand in the double-stranded RNA molecule (dsRNA) [45,46,47]. The apparent contradiction between the substrate preference of RNase III proteins and the ssRNA degradations products observed in the NCRIP supposed the basis for the thorough study of this new family of RNase III proteins related to R3B2.

The recent purification of R3B2 protein of *M. lusitanicus* revealed that the RNase uses both dsRBDs to bind either ssRNA or dsRNA in a sequence-independent manner [44]. These results suggest an additional protein that can interact with R3B2 and determine the targeted mRNAs regulated by the NCRIP. Interactions with RNase III proteins to manage or modulate their function are observed from plants [48] to animals [35]. Surprisingly, as it was hypothesized in early works, R3B2 exclusively degrades ssRNA in vitro despite the presence of a RIIID and its dimerization pattern from the RNase III family [44]. The analysis of R3B2 activity revealed that the requirement of an ssRNA substrate relies on the RIIID. Mutation of the RIIID of R3B2 disrupts its catalytical activity while the substitution of the RIIID with a canonical domain restores the ability of the hybrid RNase to process dsRNA. Conversely, despite the low aminoacidic conservation, the crystallographic structure of the RIIID of R3B2 shows a similar organization resembling the canonical RNase III proteins [44]. Striking by its absence either in R3B2 or the homologs of Mucorales, it is the lack of the linker α5/α6 which acts as RNA binding motif in the canonical RIIIDs [49].

Nevertheless, the superposition of the RIIID dimer of R3B2 with other well-characterized revealed a narrowed catalytic center. Although the observation of the RIIID structure interacting with RNA is necessary, the relative arrangement of the catalytic dimer suggests steric hindrances to the entrance of dsRNA, which could explain the inability of R3B2 to cleave dsRNA. Further research should address this question, given that the purification and analysis of the homologous RNases from other mucormycosis causing agents, as *Rhizopus microsporus* and *Lichtheimia corymbifera*, exhibited the same specificity against ssRNA [44]. Thus, it is tempting to speculate functional conservation of the NCRIP in Mucorales beyond *M. lusitanicus*.

The same in vitro analysis revealed that the second dsRBD (dsRBD2) could modulate the catalytical activity of R3B2 [44]. While a truncated version of R3B2 without dsRBD2 retains the RNase activity, the mutation of the domain produces a severe loss of activity. In the same sense, limited proteolysis of the full-length R3B2 shows that the region of the dsRBD2 is flexible and accessible for proteases, being a good candidate for protein-protein interactions like other dsRBDs [50] to regulate the RNase activity. Therefore, not just the study of the activity but also its regulation supposes promising approaches as therapeutic targets for developing new antifungal drugs. A relevant mechanism for the pathogenic potential of Mucorales organized around an unusual RNase exclusive of these fungi has been described. The thorough study of this evolutive peculiarity supposes a new way to the specific and effective treatment of mucormycosis.

## 4. Interaction between NCRIP and the Canonical RNAi Pathways: Regulation of the Epimutational Antifungal Drug Resistance

The NCRIP negatively regulates the generation of antifungal drug resistant epimutants through its control over the canonical RNAi mechanism [26]. The endogenous canonical pathway constitutively produces a particular class of esRNAs, the ex-siRNAs, which regulate hundreds of target mRNAs [22]. Recently, a subpathway of this endogenous canonical RNAi mechanism has been discovered: the epimutational pathway [19]. This epimutational pathway produces specific ex-siRNAs in response to an external stimulus. The specific ex-siRNAs are produced only from the key target genes involved in response to the external stimulus. The silencing of these target genes implies a transient and unstable adaptation to the new external conditions. The specific ex-siRNAs are mitotically passed on to the descendant epimutants as long as the external conditions are maintained. The silencing of the target genes and the production of ex-siRNAs stop when the external stimulus disappears [19].

### 4.1. Discovery of the Epimutational RNAi Pathway

The epimutational pathway was discovered by studying the effects of the antifungal compound FK506 in the fungus *M. lusitanicus* [19]. This pathogenic fungus grows as hyphae in the presence of oxygen and as a yeast when high concentrations of CO_2_ and a reducing sugar are present [51]. The antifungal effects of FK506 are a consequence of blocking the activity of the calcineurin pathway. FK506 forms a complex with a prolyl isomerase known as FKBP12 (coded by the *fkbA* gene), and this complex inhibits the protein calcineurin [52,53,54]. This inhibition represses the hyphal growth of *M. lusitanicus*, enforcing only the yeast form. The yeast phase growth is avirulent, and only the mycelial form can cause mucormycosis [9,52]. The antifungal drug resistance mediated by RNAi was observed after the exposure to FK506 for several days, which resulted in the emerging of FK506-resistant mycelia from the yeast colony perimeter. When these resistant isolates were sequenced, 70% of them presented the expected mendelian mutations in the *fkbA* gene or the genes *cnaA* and *cnbR* (encoding the two subunits of the calcineurin protein). However, the remaining 30% of resistant isolates did not present any mutation that could explain their antifungal drug-resistance phenotype (from this time on, these isolates were called epimutants) [19]. Besides, these resistant isolates showed an unstable phenotype that could revert to the wild type after growing on drug-free plates. These two observations suggested that an RNAi-based mechanism could be involved in the generation of these epimutants. To test this hypothesis, *fkbA* mRNA expression and its corresponding protein were examined in the epimutants, resulting in a complete absence of both. When the same analysis was done in epimutants grown in drug-free plates for several generations, both mRNA and protein expression were restored. The conclusive evidence proving the role of the RNAi pathway in this new antifungal mechanism was the detection of high amounts of sRNAs corresponding to the *fkbA* sequence [19].

Further investigations dissected the canonical RNAi pathway machinery, studying its role in this new antifungal resistance mechanism. In these studies, the frequency of epimutants production was analyzed in the mutants *dcl1-, dcl2-, ago1-, ago2-, ago3-, rdrp1-,* and *rdrp2-* [19]. The genes *ago2* and *ago3* had no role in the epimutational pathway. The production of epimutants was entirely lost in the mutants *dcl2-, ago1-,* and *rdrp2-,* evidencing their essential role in the canonical core of the epimutational pathway. Although the gene *dcl1* is expendable in the siRNA pathway, the frequency of epimutants production was also strongly reduced in the mutant *dcl1-,* indicating that *dcl1* and *dcl2* work together in this new epimutational pathway. The gene *rdrp2* was crucial in the epimutational pathway, though it has an accessory role in the amplification of the signal in the other two canonical pathways (siRNAs and ex-siRNAs pathways). However, the most intriguing difference among the three pathways was the role of RdRP1. The lack of *rdrp1* did not impair the production of epimutants, and surprisingly, the frequency of epimutants production raised from 30% to 80%. These results indicated a repressing activity of RdRP1 over the epimutational pathway [19].

After discovering the antifungal resistance against FK506 by the epimutational pathway, the activity of this new mechanism was examined in other target genes and antifungal compounds [55,56]. This study analyzed the resistance to the antifungal compound 5-fluoroorotic acid (5-FOA) by specific silencing of the genes *pyrF* and *pyrG*. These two genes are involved in the pathway of uridine synthesis, and their activity transforms the compound 5-FOA into a toxic derivative. Thus, the lack of any of these genes confers 5-FOA resistance in *M. lusitanicus*. Following a similar analysis of the FK506 resistant isolates, strains resistant to 5-FOA were found without any mutations in *pyrF* or *pyrG*. The study of the sRNome in these new epimutants showed sRNA production targeting *pyrF* and *pyrG*. The phenotypical analyses showed reversion to drug sensitivity after growing in a drug-free medium. These results demonstrated the capacity of *Mucor* to develop resistance to a variety of antifungal agents [55].

The epimutant generation and antifungal drug resistance processes were also studied using an in vivo murine model [56]. In this model, mice were infected with epimutant strains resistant to the antifungal agent FK506. The analysis of these mice showed that epimutant-induced drug resistance was stable in vivo in different organs and tissues, though reversion to drug sensitivity was more frequent in epimutants recovered from the brain compared to those recovered from other organs. More interestingly, the infection with a wild-type strain showed an increased epimutation rate in the strains recovered from the brain compared to other organs when the harvested spores were exposed to FK506.

### 4.2. NCRIP Represses the Epimutational RNAi Pathway

The surprising RdRP1 role inhibiting the epimutational pathway was unveiled before discovering NCRIP and its dependence on RdRP1 and R3B2 [19]. Once NCRIP was found, the issue to resolve was its putative role regulating the epimutational pathway. In this sense, the new investigations focused on the study of mutants in the *r3b2* and *rdrp3* genes [26].

Independent mutants in *r3b2* were incubated in the presence of FK506. After a few days, the fungus developed epimutational resistance to the drug in almost 80% of the isolates, precisely the same results obtained with *rdrp1* mutants in previous studies. These findings showed that the machinery necessary to keep NCRIP active is simultaneously repressing the activity of the epimutational pathway, establishing the first regulatory link between both pathways [26].

The antagonistic roles of RdRP1 and RdRP2 in the epimutational pathway suggest an unlikely collaboration in the machinery of NCRIP [36]. Thus, the existence of a third RdRP in *M. lusitanicus* was suggested. The gene *rdrp3* was discovered by its sequence similarity to the RdRP2 protein of *M. lusitanicus*. Immediately, its role in NCRIP was tested in several independent mutants by expression analyses of known mRNA targets of NCRIP [26]. Mutants in *rdrp3* showed an increase in the expression of these marker genes, similarly to *rdrp1* and *r3b2* mutants, indicating its essential role to keep NCRIP active. RdRP3 is not necessary to initiate or amplify the transgene-induced RNAi pathway, which was observed after transforming mutants with different triggering molecules. Regarding the epimutational pathway, mutations in *rdrp3* increased the generation of epimutants in FK506-medium to >80%. These results indicated that *rdrp3* is also essential to keep the repressing activity of NCRIP over the epimutational pathway [26].

### 4.3. Other Interactions among the Different RNAi Pathways of M. lusitanicus

The fact that mutations in any essential gene involved in NCRIP increases the production of epimutants was the first evidence of a canonical RNAi pathway regulated by NCRIP. These results led to new studies searching for other interactions among the different RNAi pathways found in *M*. *lusitanicus.* Studies in *N. crassa* identified two components of the transgene-induced RNAi pathway known as quelling induced protein (*qip*) and a Sad-3-like helicase (*rnhA*) [57,58,59]. QIP is an enzyme with exonuclease activity related to the RNAi pathway because of its interaction with Argonaute during the RISC complex assembly in *N. crassa*. In *M. lusitanicus*, there is an ortholog containing the three exonuclease characteristic motifs of the DEDDh superfamily, also harboring the essential conserved amino acids. The corresponding *qip*- null mutant was tested with different RNAi triggers, observing that this protein plays a critical role in the transgene-induced RNAi pathway, similar to the function previously demonstrated in *N. crassa* [26]. Regarding the epimutational pathway, independent mutants in the *qip* gene were unable to generate epimutant strains resistant to FK506, indicating an essential role in the epimutational RNAi pathway. No studies dissected the role of *qip* in the NCRIP machinery. However, the indispensable role of *qip* in the epimutational pathway and the Argonaute-independence of NCRIP strongly suggest a non-critical role of this gene in NCRIP.

RNA helicases have also been involved in the RNAi mechanism of fungal models [59]. Using homology analysis, a putative RNA helicase was found in *M*. *lusitanicus* and named *rnhA* [26]. The encoded protein contains the conserved domains such as UvrD-like helicase C-terminal domain, DEAD-like helicase superfamily domain, and a zinc-binding domain. Following the same analyses done with *qip*, mutants in *rnhA* showed a minor and expendable role in the transgene-induced RNAi pathway. However, and similarly to *qip*, *rnhA* was essential for the epimutational pathway [26].

## 5. Role of NCRIP in the Integrity and Defense of the Genome

Despite NCRIP negatively regulates the epimutational pathway, its most specific component, R3B2, is also necessary for the transgene-induced RNAi silencing. The role of R3B2 in the transgene-induced RNAi pathway was confirmed after transforming *r3b2* mutants with different exogenous triggering molecules. The frequency of silenced transformant was reduced in the *r3b2* mutants, indicating that R3B2 is critical for efficient silencing of exogenous molecules [23]. The minor role of R3B2 in the canonical RNAi pathways is likely related to a putative interaction of the Dicer proteins, although its function in these pathways is still unknown [60]. The discrimination of R3B2 between the canonical and non-canonical pathways is also undetermined. The proposed hypothesis suggests a likely collaboration of R3B2 with either Dicer or the RdRP proteins to participate in the canonical RNAi mechanism or NCRIP, respectively.

The role of R3B2 in the transgene-induced and the endogenous canonical RNAi pathways is independent of its duties in the NCRIP mechanism. However, recent studies have found a new role of R3B2 and the other components of NCRIP in the negative regulation of the expression of centromeric retrotransposons [27,61]. In these studies, a particular type of centromeres (mosaic centromeres) was identified in Mucorales. One of these centromeres features is the presence of autonomous repeats comprising two sequential open reading frames with similar sequence and architecture compared to non-LTR retrotransposon belonging to the LINE1 clade. These retrotransposons precisely localize in the pericentric regions arranged as inverted repeats that have been proved essential for centromeric function in other fungal organisms [51,62]. It is known from previous studies that RNAi represses expression and inhibits transposition of retrotransposable elements in several organisms [63,64]. In *M. lusitanicus*, the role of the canonical RNAi machinery was found essential when the expression of these retrotransposable elements was studied in the wild type and *dicer2* and *ago1* mutants. Thus, the pericentric regions had the expression of retrotransposons reduced in the wild type and, conversely, these regions expressed sRNAs. However, the mutants in essential components of the RNAi machinery exhibited high mRNA levels and decreased sRNAs production. These results indicated a protective role of the canonical RNAi pathway against the deleterious activity of these mucoralean transposons [61]. Later, new studies investigated the role of NCRIP in the regulation of these mucoralean retrotransposons. The lack of NCRIP in mutant strains triggered a strong overaccumulation of sRNAs and a complete depletion of mRNAs compared with wild-type strains [27]. These results indicated a negative regulatory role of NCRIP over the canonical repression of transposons, similar to the repression of NCRIP over the epimutational RNAi pathway.

## 6. The NCRIP Mechanism Regulates Virulence in Mucorales

The lack of an active NCRIP mechanism results in misregulation of the genetic response of mucoralean spores during the confrontation with macrophages, the first stage of the infection [27,65]. The studies confronting the sRNAs produced explicitly by the NCRIP mechanism with the genome sequence of *M. lusitanicus* found that this pathway regulates many genes [23]. Therefore, it is expected a pleiotropic role of NCRIP in the fungal physiology of Mucorales, controlling diverse processes. In fact, the phenotypic analysis of strains lacking the NCRIP activity confirmed the role of this mechanism in complex cellular processes such as the response to oxidative stress and the development of zygospores during sexual development [23]. The phenotype affecting the production of zygospores can be related to the regulatory role of NCRIP over the canonical RNAi pathway during the control of the centromeric transposons. In *Cryptococcus neoformans*, RNAi-related mechanisms protect the genome from transposon activity during mating, securing the genomic integrity of the progeny [66]. The second phenotype, the misregulated response to oxidative stress, might be related to a functional role of NCRIP in a natural scenario where this stress is activated, for instance, during the oxidative attack of macrophages after phagocytosis. Thus, thorough studies investigated the role of NCRIP during the interaction of macrophage and mucoralean spores. These reports found that the genetic response of *M. lusitanicus* during the interaction with macrophages showed hundreds of misregulated genes. An exhaustive study of this misregulated response indicated that the role of NCRIP is to keep repressed a pool of phagocytosis-related genes during non-stressful conditions when there is not an interaction with macrophages. Mutant strains with an inactive NCRIP are blinded for the oxidative response, activating the genetic program against this stress when it is not present. A properly regulated genetic response to escape from macrophages is critical for the infection. In consequence, NCRIP mutants show avirulent phenotypes in murine models [27].

## 7. Conclusions

NCRIP is a new RNAi pathway with non-canonical machinery dependent on RdRPs and a novel RNase known as R3B2. Its main activity is to regulate the expression of target mRNAs by their degradation during specific non-stressful conditions. The most peculiar feature of NCRIP is its independence on Dicer and Argonaute proteins and their substitution with the enzyme R3B2. This novel RNase type III has a particular degrading activity because it exclusively degrades ssRNA, unlike other RNases type III that only cut dsRNA. The simplicity of the NCRIP machinery and its independence on Dicer and Argonaute proteins led to propose this non-canonical pathway as an evolutionary link between the bacterial RNA degrading pathways and the complex eukaryotic RNAi pathways [23,67]. Its machinery is similar to bacterial RNA degradation pathways, but its complex regulatory functions relate to the complex canonical RNAi pathways in eukaryotes. The regulatory functions of NCRIP include the repression of the epimutational pathway and the generation of antifungal resistant strains, the repression of the canonical RNAi in the control of transposon expression, the regulation of the fungal genetic response during phagocytosis, and the multifactorial process of virulence in Mucorales. The critical role of NCRIP in virulence and its function in regulating the antifungal resistance response make this pathway a key element in the development of mucormycosis and its treatment. Moreover, its specific conservation in Mucorales makes this non-canonical pathway an ideal target for developing new therapies against mucormycosis.
